# Factors Associated With Specialists’ Intention to Adopt New Behaviors After Taking Web-Based Continuing Professional Development Courses: Cross-sectional Study

**DOI:** 10.2196/34299

**Published:** 2022-06-02

**Authors:** Lysa Bergeron, Simon Décary, Codjo Djignefa Djade, Sam J Daniel, Martin Tremblay, Louis-Paul Rivest, France Légaré

**Affiliations:** 1 VITAM - Centre de recherche en santé durable Centre intégré universitaire de santé et de services sociaux de la Capitale-Nationale Quebec, QC Canada; 2 Department of Social and Preventive Medicine, Faculty of Medicine Université Laval Quebec, QC Canada; 3 School of Rehabilitation, Faculty of Medicine and Health Sciences Université de Sherbrooke Sherbrooke, QC Canada; 4 Direction du Développement Professionnel Continu Fédération des Médecins Spécialistes du Québec Montreal, QC Canada; 5 Department of Pediatric Surgery McGill University Montreal, QC Canada; 6 Department of Mathematics and Statistics, Faculty of Science and Engineering Université Laval Quebec, QC Canada; 7 Department of Family Medicine and Emergency Medicine Faculty of Medicine Université Laval Quebec, QC Canada

**Keywords:** continuing professional development, CPD-Reaction, behavioral intention, medical specialists, continuing professional development, web-based training, medical education, education, physician, psychosocial, online course

## Abstract

**Background:**

Web-based continuing professional development (CPD) is a convenient and low-cost way for physicians to update their knowledge. However, little is known about the factors that influence their intention to put this new knowledge into practice.

**Objective:**

We aimed to identify sociocognitive factors associated with physicians’ intention to adopt new behaviors as well as indications of Bloom’s learning levels following their participation in 5 web-based CPD courses.

**Methods:**

We performed a cross-sectional study of specialist physicians who had completed 1 of 5 web-based CPD courses offered by the Federation of Medical Specialists of Quebec. The participants then completed CPD-Reaction, a questionnaire based on Godin’s integrated model for health professional behavior change and with evidence of validity that measures behavioral intention (dependent variable) and psychosocial factors influencing intention (n=4). We also assessed variables related to sociodemographics (n=5), course content (n=9), and course format (eg, graphic features and duration) (n=8). Content variables were derived from CanMEDS competencies, Bloom’s learning levels, and Godin’s integrated model. We conducted ANOVA single-factor analysis, calculated the intraclass correlation coefficient (ICC), and performed bivariate and multivariate analyses.

**Results:**

A total of 400 physicians participated in the courses (range: 38-135 physicians per course). Average age was 50 (SD 12) years; 56% (n=223) were female, and 44% (n=177) were male. Among the 259 who completed CPD-Reaction, behavioral intention scores ranged from 5.37 (SD 1.17) to 6.60 (SD 0.88) out of 7 and differed significantly from one course to another (*P*<.001). The ICC indicated that 17% of the total variation in the outcome of interest, the behavioral intention of physicians, could be explained at the level of the CPD course (ICC=0.17). In bivariate analyses, social influences (*P*<.001), beliefs about capabilities (*P*<.001), moral norm (*P*<.001), beliefs about consequences (P<.001), and psychomotor learning (*P*=.04) were significantly correlated with physicians’ intention to adopt new behaviors. Multivariate analysis showed the same factors, except for social influences and psychomotor learning, as significantly correlated with intention.

**Conclusions:**

We observed average to high behavioral intention scores after all 5 web-based courses, with some variations by course taken. Factors affecting physicians’ intention were beliefs about their capabilities and about the consequences of adopting new clinical behaviors, as well as doubts about whether the new behavior aligned with their moral values. Our results will inform design of future web-based CPD courses to ensure they contribute to clinical behavior change.

## Introduction

Improving patient outcomes requires that health professionals constantly adjust their practices in light of new evidence. Continuing professional development (CPD) is one of the most common strategies for achieving this, and indeed is a requirement for continued practice in many countries, including Canada [1]. The use of web-based CPD increased 10-fold from 2002 to 2008 in the United States and continues to grow rapidly [2], paralleling the increasing use of other web-based tools by health professionals [3]. A combination of the high costs of in-person CPD and the sanitary measures imposed during the COVID-19 pandemic have accelerated this increase in web-based CPD [4,5]. It is not clear whether web-based CPD has a real impact on clinical practice [6-9] or if physicians want CPD delivered this way [10]. However, in times of pandemic there is little choice, and some of the advantages of distance learning have been highlighted in this context [11]. High-quality CPD courses should translate the evidence presented not only into new awareness, but also into new practices; yet most studies only evaluate their impact on clinical practice using measures of satisfaction and changes in knowledge [6,12,13]. Several meta-analyses on the impact of CPD on physician performance have recommended that new research should focus less on whether CPD is effective and more on why it is effective [14]. This requires a better understanding of the theory-based mechanisms underlying the impact of web-based CPD courses on clinical practice [15]. Future courses could then be based on these evidence-based and theory-informed mechanisms.

Sociocognitive theories describe these mechanisms by identifying key variables and the interrelationship of determinants in predicting health behaviors [16]. Studies based on such theories provide the empirical evidence to guide many behavior-change interventions. Learning is based not only on absorbing information, but on other factors that produce social behaviors, such as social modeling and a personal sense of control [16-19]*.* To ensure that CPD courses lead to physicians adopting the desired behavior in clinical practice, it is essential that they be informed by sound, theory-based factors known to influence the adoption of a given behavior [20-22]. According to Godin’s integrated model for health professional behavior change [17], behavioral intention is the central factor influencing the adoption of a given behavior. In turn, this intention is influenced by a number of other sociocognitive factors. Incorporating these modifiable sociocognitive factors in the design of CPD has proven acceptable and feasible [23] and holds great promise for improved clinical practices [15,24].

Godin’s comprehensive list of these factors, gathered from evidence produced by multiple studies in numerous domains, informed our CPD-Reaction tool, designed to assess behavioral intention after CPD activities [19,25]. The questionnaire consists of 12 items related to intention and 4 of the following influences on intention: (1) social influence (perception of approval or disapproval by persons significant to the individual regarding the adoption of the behavior); (2) beliefs about capabilities (belief that one is capable of performing the behavior); (3) moral norm (feeling of personal obligation regarding the adoption of the behavior); and (4) beliefs about consequences (perception that the behavior will have harmful or beneficial effects). For CPD to result in adopting a new clinical or organizational practice, “deep” learning also needs to occur [26]. Many CPD developers use Bloom’s taxonomy to design the learning objectives of CPD activities, which also provides measures for their effects [27]. Bloom’s taxonomy is related to Kirkpatrick’s model, one of the best-known models for analyzing and evaluating the results of training programs [28]. Bloom’s taxonomy provides additional detail by defining 3 domains of learning, which are affective, psychomotor, and cognitive. Affective learning relates to attitudes, psychomotor to physical skills, and cognitive to six learning levels, each of increasing “depth” or complexity [29].

Therefore, to address the lack of theory-informed assessment of CPD activities, we aimed to identify sociocognitive factors associated with physicians’ intention as well as indications of Bloom’s learning levels following their participation in 5 web-based CPD courses.

## Methods

### Study Design

We performed a cross-sectional study of a convenience sample of specialist physicians who had completed 1 of 5 different web-based CPD courses [30]. We report data according to the STROBE (Strengthening the Reporting of Observational Studies in Epidemiology) reporting guidelines for cross-sectional studies [31]. Data were collected between November 2015 and April 2019 following completion of the CPD courses by participants using a web-based interactive platform (MÉDUSE) designed by the Federation of Medical Specialists of Québec (FMSQ) [32]. The FMSQ consists of 35 medical associations and represents 59 medical specialties in the province of Quebec. Its members include more than 10,000 medical specialists [33].

### Ethics Approval

Approval for this study was obtained from the research ethics boards of the Centre intégré universitaire de santé et de services sociaux (CIUSSS) de la Capitale-Nationale (Project 2020-1889_SPPL).

### Study Participants

To be eligible, the physicians had to have completed (1) one of the 5 available FMSQ CPD web-based courses and (2) the CPD-Reaction questionnaire.

### CPD Courses

The 5 web-based courses were all accredited by the Royal College of Physicians and Surgeons of Canada [34] and targeted the following five behaviors: (1) to adapt the frequency of cytological exams for gynecological patients 25-45 years old to new human papillomavirus recommendations; (2) to use recommended lung cancer treatment and monitoring algorithms; (3) to use a systematic leadership approach in community health endeavors (eg, preventing instances of suicide from a bridge in Montreal); (4) to respect best practices in record keeping; and (5) to identify patients who meet the criteria for identifying a potential organ donor. Courses were free of commercial support (paid by FMSQ members’ annual contributions). They were secured and accessible 24/7 by Quebec specialist physicians. The courses lasted from 90 to 120 minutes, and participants could stop or reinitiate courses at any time. Course objectives were based on Godin’s integrated model for health professionals’ behavior and aimed to encourage physicians to adopt new behaviors (or cease old ones). Each targeted behavior was designed according to 3 of the TACT principles: “target,” “action,” and “context” (“time” was excluded, as the targeted behaviors were not dependent on a specific time frame) [35]. The courses were also designed to develop core competencies as described in the CanMEDS Competency Framework [36]. CanMEDS is a framework created by the Royal College to ensure that CPD courses, regardless of their specialist content, allow physicians to develop one or more of the following core roles: medical expert, communicator, collaborator, leader, health advocate, scholar, or professional. We inserted the relevant learning objectives into each of the 5 CPD-Reaction questionnaires and attached them to the end of each respective course (Figure S1 in Multimedia Appendix 1).

### Data Collection Procedure

Data were collected in 2 separate databases by the FMSQ, one for the sociodemographic variables of those attending each course (henceforth referred to as “participants”) and another for those who had completed CPD-Reaction (henceforth referred to as “respondents”). Individual participant sociodemographic data could not be linked to individual respondent CPD-Reaction questionnaire scores and were analyzed at the level of the CPD course. For sociodemographic variables and variables collected at the course level (content and format of the CPD courses), the same values were then attributed to all respondents in the same course.

#### At the Level of Participants

##### Psychosocial Determinant Variables (1 Dependent Variable and 4 Independent Variables)

The CPD-Reaction is a self-administered questionnaire based on sociocognitive theories of behavioral change. The questionnaire had been developed and validated earlier with participants in 18 different CPD activities and had a Cronbach α ranging from .77 to .85 [22,25]. The CPD-Reaction questionnaire consists of 12 items grouped into the five following constructs: (1) behavioral intention (dependent variable; 2 items); (2) beliefs about capabilities (3 items); (3) social influences (3 items); (4) beliefs about consequences (2 items); and (5) moral norm (2 items). The specific clinical behavior targeted by the CPD course is inserted into each item of the questionnaire. There is no overall score for CPD-Reaction. The score for each construct is computed as the average of each item (Likert scale of 1, which is low, to 7, which is high), except for social influence, which is rated on a Likert scale of 1 to 5 [37]. Thus, a moral norm score of 7, for example, indicates that the respondent feels a strong obligation to adopt this behavior, while a score of 1 for beliefs about capabilities indicates that the respondent does not feel confident in their ability to adopt the behavior [37]. All physicians who completed CPD courses were invited to fill out the CPD-Reaction questionnaire afterward.

#### At the Level of the CPD Courses

##### Participant Profile (Sociodemographic) Variables (5 Independent Variables)

All participants (n=400) provided information about their age, number of years in practice, sex (female or male), their medical association (clinical area), and administrative region.

##### Characteristics of Course Content (9 Independent Variables)

Two coders independently noted the presence of slides in each CPD course in which they could identify the following elements: (a) targeting of a CanMEDS role—medical expert, communicator, collaborator, leader, health advocate, scholar, or professional (when more than one role was targeted, the reference category was “not applicable”); (b) Bloom’s learning levels; and (c) constructs of Godin’s theoretical framework for the study of health care professionals’ behavior and intention [17,27,36].

##### Characteristics of Course Format (8 Independent Variables)

Informed by literature on presentation of material for optimal learning [38,39], 2 coders independently assessed the presence of the following factors: use of virtual characters, use of a reflective approach, duration of the course, presence of nonfunctional links to references, presence of slides with a video of a health professional (opinion leader), presence of slides with a figure or a diagram, presence of slides with a quiz, and presence of women on the scientific committee for the course development.

### Data Analysis

The data set had a hierarchical structure consisting of 2 levels, which were respondents and CPD courses. Individual participant sociodemographic data were in a distinct database and could not be linked to individual respondent CPD-Reaction questionnaire scores. Therefore, analysis focused primarily on variables at the level of CPD courses. These variables were only retained if they could be collected for all 5 courses. All variables retained at the course level had fewer than 1% missing values. At the respondent level (n=259), only the dependent variable, intention, and the 4 independent psychosocial variables (social influences, beliefs about capabilities, moral norm, and beliefs about consequences) were accessible and analyzed. At the CPD course level (n=5 CPD courses), data analyzed included the 5 sociodemographic variables of participants, 8 course format variables, and 9 course content variables.

We used descriptive statistics and frequency counts to describe all variables. We performed an ANOVA single-factor analysis to assess whether the topic of the courses had an impact on intention. We also computed the intraclass coefficient (ICC) to assess the percentage of variance in behavioral intention and its psychosocial determinants attributable to the CPD course [40]. We performed exploratory bivariate analysis using Spearman correlations for each one of our independent variables at the level of the CPD courses to assess their association with physicians’ intention scores. Lastly, we performed bivariate and multivariate analysis on the 4 psychosocial determinants of intention at the respondent level (n=259) to explore their impact on intention scores. We used a linear model and introduced a random effect for the CPD courses. A threshold of .05 was set for statistical significance. We verified all assumptions for the linear regression model [41]. All analyses were performed with SAS, version 9.4 (SAS Institute Inc).

## Results

### Characteristics of the Participants

Table 1 shows the characteristics of participants from across Quebec attending each course.

**Table 1:** Profile of the participants.

Variables			Courses^a^										
			1		2		3		4		5		All courses
Participants, n (%)			96 (24)		60 (15)		71 (18)		135 (34)		38 (9)		400 (100)
Age (years), mean (SD)			48 (11)		49 (11)		51 (13)		52 (12)		46 (12)		50 (12)
**Gender, n (%)**													
	Female	70 (73)		20 (33)		43 (61)		74 (55)		16 (42)		223 (56)	
	Male	26 (27)		40 (67)		28 (39)		61 (45)		22 (58)		177 (44)	
Main clinical area for each course, n (%)			67 (70)^b^		24 (40)^c^		11 (15)^d^		23 (17)^e^		7 (18)^f^		87 (22)^b^
Most frequent administrative area of main practice site, n (%)			18 (19)^g^		18 (30)^h^		14 (20)^h^		35 (26)^g^		6 (16)^h^		77 (19)^g^
Years of practice, mean (SD)			19 (13)		19 (13)		20 (14)		22 (14)		14 (13)		19 (14)

^a^Course details: behaviors used in the questionnaire for each course; course 1—to adapt the frequency of cytological examinations according to new recommendations; course 2—to use lung cancer treatment and monitoring algorithms; course 3—to use a systematic leadership approach; course 4—to respect good practices in record keeping; and course 5—to identify patients who meet the criteria for identifying a potential organ donor.

^b^Obstetrics and gynecology.

^c^Pneumology.

^d^Preventive medicine.

^e^Psychiatry.

^f^Anesthesiology.

^g^Montérégie.

^h^Montreal.

### CPD Course Characteristics

Table 2 shows details of course characteristics, including course formatting, content, presence of Godin’s constructs, and Bloom’s learning levels. Three courses lasted 90 minutes and 2 lasted 120 minutes. Two out of 5 courses focused on the CanMEDS role of medical expert, 1 on the role of leader, 1 on the role of professional, and 1 on several of the roles at once (classified as “not applicable”). Moreover, 3 course characteristics showed no variability—all contained slides with a quiz (format variable), slides on beliefs about capabilities, and slides on role and identity (content variables; Table 2).

**Table 2:** CPD^a^ course characteristics.

Variables			Course^b^				
			1	2	3	4	5
**Characteristics of course format**							
	Virtual character use in course		Yes	No	Yes	Yes	No
	Use of a reflective approach		Yes	No	No	Yes	Yes
	Duration, min		90	120	120	90	90
	Presence of nonfunctional references		Yes	No	No	No	Yes
	Presence of slides with a video of health professional—leader opinion		No	No	No	No	Yes
	Presence of slides with a figure or a diagram		No	Yes	Yes	Yes	Yes
	Presence of slides with a quiz		Yes	Yes	Yes	Yes	Yes
	Presence of women on the scientific committee		No	—^c^	—	Yes	Yes
**Characteristics of course content**							
	Main CanMED role		Medical expert	Medical expert	Leader	Professional	N/A^d^
	**Presence of slides per constructs of Godin’s integrated model for health professional behavior change**						
		Intention	Yes	Yes	Yes	No	Yes
		Social influences	No	No	Yes	No	Yes
		Beliefs about capabilities	Yes	Yes	Yes	Yes	Yes
		Role and identity	Yes	Yes	Yes	Yes	Yes
		Beliefs about consequences	Yes	No	Yes	Yes	Yes
	**Presence of which level of Bloom’s taxonomy**						
		Cognitive	Yes	Yes	Yes	Yes	No
		Affective	No	No	No	No	Yes
		Psychomotor	No	No	No	Yes	Yes

^a^CPD: continuing professional development.

^b^Course details: course 1—to adapt the frequency of cytological exams for gynecological patients 25-45 years old to new Human Papillomavirus recommendations; course 2—to use recommended lung cancer treatment and monitoring algorithms; course 3—to use a systematic leadership approach in community health endeavors (eg, preventing suicides from a bridge in Montreal); course 4—to respect best practices in record keeping; and course 5—to identify patients who meet the criteria for identifying a potential organ donor.

^c^Not available.

^d^N/A: not applicable.

### CPD-Reaction Questionnaires Scores and ICC

Of the 400 participants, 259 (65%) respondents fully completed the CPD-Reaction questionnaire. Table 3 shows details of respondents’ mean intention and psychosocial determinants and SD scores for each course. The behavioral intention score was medium to high and varied depending on the course undertaken (intention score between 5.37, SD 1.17 and 6.60, SD 0.88). ANOVA analysis of the variable intention showed significant differences between courses (*F* value=12.50, *P*<.001). The ICC (0.17) indicated that 17% of the total variation in the behavioral intention of physicians to adopt new behaviors could be explained at the level of the course in which they had registered. Some courses showed significantly different means of intention, that is, respondents shared more intracourse similarities than extracourse similarities (ie, within CPD courses vs between courses).

**Table 3:** CPD-Reaction questionnaire mean scores and ICC^a^.

Variables			Courses^b^											Mean (SD)			ICC
			1		2		3		4		5						
Number of respondents, n (%)			53 (20)		44 (17)		63 (24)		61 (24)		38 (15)		N/A^c^			N/A	
**Psychosocial determinants^d^, mean (SD)**																	
	Intention	6.22 (1.15)		6.22 (0.87)		5.37 (1.17)		6.57 (1.11)		6.60 (0.88)		6.15 (1.16)			16.8		
	Social influences	5.44 (0.97)		5.41 (1.12)		4.39 (0.98)		5.17 (0.95)		5.16 (1.33)		5.07 (1.12)			13.0		
	Beliefs about capabilities	6.28 (0.80)		6.22 (0.75)		5.07 (0.81)		6.16 (0.89)		6.25 (1.11)		5.94 (0.99)			25.9		
	Moral norm	6.58 (0.66)		6.35 (0.82)		5.94 (0.93)		6.61 (1.11)		6.68 (0.80)		6.41 (0.93)			8.98		
	Beliefs about consequences	6.28 (0.94)		6.30 (0.94)		5.99 (0.91)		6.72 (0.47)		6.68 (0.77)		6.38 (0.86)			10.9		

^a^ICC: intraclass correlation coefficient.

^b^Course details: course 1—to adapt the frequency of cytological exams for gynecological patients 25-45 years old to new Human Papillomavirus recommendations; course 2—to use recommended lung cancer treatment and monitoring algorithms; course 3—to use a systematic leadership approach in community health endeavors (eg, preventing suicides from a bridge in Montreal); course 4—to respect best practices in record keeping; and course 5—to identify patients who meet the criteria for identifying a potential organ donor.

^c^N/A: not applicable.

^d^Score range 1-7.

### Factors Associated With Physicians’ Intention to Adopt a New Behavior

Only one of the course variables, psychomotor learning level (Bloom’s taxonomy), was significantly associated with the physicians’ intention to change their behavior, and this was the case in all 5 courses (*R*=0.89, *P*=.04) (data not shown). Bivariate regression analysis of psychosocial determinants showed that all 4 variables were significantly associated with intention (*P*<.001) (Table 4). Multivariate regression analysis of the same variables showed 3 out of the 4 were significantly correlated with intention, namely beliefs about capabilities (0.49, *P*<.001), moral norm (0.37, *P*<.001), and beliefs about consequences (0.40, *P*<.001) (Table 4). When we analyzed the courses separately, we found similar results (Multimedia Appendix 2).

**Table 4:** Bivariate regression analysis and multivariate regression analysis of psychosocial determinants associated with intention to adopt a clinical behavior (n=259 respondents).

Variables		β	95% CI	*P* value
**Bivariate regression analysis**				
	Social influences	.42	0.31 to 0.53	<.001
	Beliefs about capabilities	.95	0.86 to 1.04	<.001
	Moral norm	.82	0.71 to 0.93	<.001
	Beliefs about consequences	.80	0.67 to 0.92	<.001
**Multivariate regression analysis**				
	Social influences	–.04	–0.12 to 0.04	.30
	Beliefs about capabilities	.49	0.37 to 0.62	<.001
	Moral norm	.37	0.27 to 0.48	<.001
	Beliefs about consequences	.40	0.30 to 0.50	<.001

## Discussion

### Principal Findings

We identified factors associated with physicians’ intention to adopt new behaviors following the completion of 5 different web-based CPD courses. Behavioral intention scores were average to high but differed significantly from one course to another. The differences between CPD courses (higher level in our hierarchical database) explained a significant proportion of this variance in intention (ICC=0.17). We saw no influence of course characteristics (content-wise or format-wise) on intention except the targeting of Bloom’s psychomotor learning level. Finally, we observed that together, beliefs about capabilities, moral norm, and belief about consequences (3 of the psychosocial variables included in Godin’s integrated model for health professional behavior change) partially explained physicians’ behavioral intentions.

### Significance and Comparison With Prior Work

First, we found that behavioral intention scores were average to high but varied by course. Some courses seemed to be associated with higher physician intention to adapt their practice compared with others. Course 3, on using a systematic leadership approach in community health endeavors, had the lowest intention score of all—although this course also targets more complex and ambiguous outcomes than the others, and leadership skills are difficult to develop in 120 minutes. We also found that the variance in intention explained by the difference in CPD courses had significant magnitude. We obtained an ICC of 0.17; thus, the intergroup variance represented 17% of the total variance. Interestingly, higher ICCs are more often seen in studies in specialty settings than in primary care studies [42]. It is possible that specialist physicians have more in common with each other, even diverse specialist physicians attending the same course, than do general practitioners. Some studies have observed that medical professional culture ensures there is more similarity than diversity within specific medical specialties [43-45].

Second, in bivariate analyses at the CPD course level, the only variable significantly correlated with the intention to adopt new behaviors was targeting Bloom’s psychomotor learning level. This level of learning, unlike the cognitive or affective levels, is more closely related to physical changes in behavior. However, this variable was not retained in our final model, suggesting that as an influence on adoption of new behaviors, it does not supersede the psychosocial variables included in the integrated model [17]. Regarding the other nonsignificant variables, previous studies have also found little significant association between sociodemographic characteristics and intention to adopt new behaviors [46,47]. Our results validate the assumption of the integrated model for health professional behavior change: modifiable psychosocial factors are the variables most likely to explain behavior change, and CPD courses should therefore focus on these factors to be more effective.

Third, we found that the 3 variables most significantly associated with intention to adopt a behavior among respondents were all psychosocial factors included in our integrated theoretical framework—beliefs about capabilities, moral norm, and beliefs about consequences (ie, their confidence about adopting the behavior, its ethical acceptability, and their perception that the behavior would be useful and beneficial). Based on our results, CPD courses should use behavior change techniques that focus on these 3 variables [48,49]. To improve beliefs about capabilities, courses could provide more experience to give participants confidence in their abilities, such as identifying barriers and management strategies, providing feedback, and encouraging monitoring of future actions (eg, noting and recording when the new behaviors have been adopted) [17,50]. To improve beliefs about consequences, courses could provide information about the proven benefits of the behavior and personalized information about its consequences. Regarding moral norm, courses could emphasize the felt obligation to adopt behaviors or help participants focus on moral considerations such as being aware of others’ needs [51]. In addition, according to Godin’s theory, when people hold two ideas that are not psychologically consistent, to reduce cognitive dissonance, they do all in their power to change them until they become consistent [17]. One way to reduce cognitive dissonance is to solicit arguments from the subject in favor of the behavior to be adopted even if they are against it [52]. While producing such arguments may cause discomfort, the subject will ultimately adjust their initial attitude to be more consistent with the arguments they fabricated in favor of the behavior. Surprising as it may seem, when we are led to act contrary to our convictions, we tend to justify our actions, and we adapt our opinions to our behavior. Other work on “provisional selves” suggests that playing a role with which one is unfamiliar, or even against which one resists, opens new moral possibilities and can help one envisage adapting one’s current role or adopting new ones [53,54]. Including this technique in a CPD course would be an interesting challenge.

### Limitations and Strengths

This was a cross-sectional study, which limited our interpretation to assuming that attending the CPD courses improved intention scores. Indeed, we are unaware if respondents already had moderate-to-high intention to adopt these behaviors before completing the CPD courses—using the CPD-Reaction questionnaire both before and after the course would have better indicated a change in intention due to the course topic. A future study with a more robust study design (eg, pre-post controlled trial) could further verify the impact of courses [55]. Moreover, to increase power, we brought data from all 5 CPD courses (each targeting a different clinical behavior) into one hierarchical data set. Although aggregating data on distinct behaviors is not always advisable [56,57], this limitation was mitigated by respect for the theory archetypes that structure the study. In addition, our sensitivity analysis (Multimedia Appendix 2) showed similar results to those obtained with the aggregated database. The literature suggests that at least 30 units at each level of analysis are needed to reach sufficient power [58]. New ways to assess CPD courses are needed as few individual CPD courses recruit hundreds of participants. Moreover, sociodemographic data collected at the group level could not be applied to the respondent level (ie, to the individual level). Inferring results of analysis at the upper level (where determinants and outcomes are related at the group [course] level) to the individual level (ecological fallacy) or the reverse (atomistic fallacy) can result in bias [59]. Finally, intention is recognized as a limited proxy for behavior. Meta-analytic syntheses have found that intention accounts, on average, for only about 25% of the variance in behavior [35,60], although finding other reliable measures of behavior is challenging [19,56]. While a 2006 review by Eccles et al [61] “provide[d] encouragement for the contention that there is a predictable relationship between the intentions of a health professional and their subsequent behaviour,” CPD activities making use of the determinants of intention as dependent variables should also integrate methods to close the intention-behavior gap such as audit and feedback, eliciting of implementation intentions (“if-then” plans), commitment to change statements, and supervision to support clinicians in following through on their intentions [62-64].

### Conclusions

Beliefs about capabilities, moral norm, and belief about consequences partially explained physicians’ behavioral intention. To address these beliefs, CPD activities could focus on building physicians’ confidence about overcoming obstacles and on strategies for helping them align moral values with new behaviors, as well as providing information about their proven benefits.

As mentioned in our previous work [12], the use of CPD-Reaction helps CPD developers reflect on the nature of their training objectives in relation to the impact they seek. This study provides insights as to how to optimize physicians’ intention to adopt a new behavior as a result of web-based CPD activities. CPD-Reaction contains the relevant theory-informed and validated items needed to assess intention and its determinants for CPD developers targeting clinical behavior change.

## Appendix

Multimedia Appendix 1 CPD-Reaction questionnaire.

Multimedia Appendix 2 Bivariate regression analysis and multivariate regression analysis of psychosocial determinants associated with intention to change a clinical behavior for each continuing professional development course (n=5). Course topic 1: to adapt the frequency of cytological exams for gynecological patients 25-45 years old to new human papillomavirus recommendations; course topic 2: to use recommended lung cancer treatment and monitoring algorithms; course topic 3: to use a systematic leadership approach in community health endeavors (eg, preventing suicides from a bridge in Montreal); course topic 4: to respect best practices in record keeping; and course topic 5: to identify patients who meet the criteria for identifying a potential organ donor.

## Abbreviations

CPD: continuing professional development
FMSQ: Federation of Medical Specialists of Québec
ICC: intraclass correlation coefficient
STROBE: Strengthening in the Reporting of Observational Studies in Epidemiology
