# Supplementary material for: Factors Associated With Specialists’ Intention to Adopt New Behaviors After Taking Web-Based Continuing Professional Development Courses: Cross-sectional Study
Source: JMIR Med Educ. 2022 Jun 2;8(2):e34299. doi: 10.2196/34299 (PMC9204572; doi:10.2196/34299)
Supplement: Multimedia Appendix 1 [file mededu_v8i2e34299_app1.pdf]

Please answer each of the following questions by indicating the number that best describes your opinion about the behavior indicated . Some of the questions may appear to be similar, but they do address somewhat different aspects of the behavior stated.

1. I intend to *[behavior]*.	Stongly disagree 1 ☐ 2 ☐ 3 ☐ 4 ☐ 5 ☐ 6 ☐ 7 ☐ Stongly agree	
2. To the best of my knowledge, the percentage of my colleagues who *[behavior]* is:	0-20% ☐ 21-40% ☐ 41-60% ☐ 61-80% ☐ 81-100% ☐	
3. I am confident that I could *[behavior]* if I wanted to.	Stongly disagree 1 ☐ 2 ☐ 3 ☐ 4 ☐ 5 ☐ 6 ☐ 7 ☐ Stongly agree	
4. *[Behavior]* is the ethical thing to do.	Stongly disagree 1 ☐ 2 ☐ 3 ☐ 4 ☐ 5 ☐ 6 ☐ 7 ☐ Stongly agree	
5. For me, *[behavior]* would be:	Extremely difficult 1 ☐ 2 ☐ 3 ☐ 4 ☐ 5 ☐ 6 ☐ 7 ☐ Extremely easy	
6. Now think about a co-worker whom you respect as a professional. In your opinion, does he/she *[behavior]*?	Never 1 ☐ 2 ☐ 3 ☐ 4 ☐ 5 ☐ 6 ☐ 7 ☐ Always	
7. I plan to *[behavior]*.	Strongly disagree 1 ☐ 2 ☐ 3 ☐ 4 ☐ 5 ☐ 6 ☐ 7 ☐ Strongly agree	
8. Overall, I think that for me *[behavior]* would be:	Useless 1 ☐ 2 ☐ 3 ☐ 4 ☐ 5 ☐ 6 ☐ 7 ☐ Useful	
9. Most people who are important to me in my profession *[behavior]*.	Stongly disagree 1 ☐ 2 ☐ 3 ☐ 4 ☐ 5 ☐ 6 ☐ 7 ☐ Stongly agree	
10. It is acceptable to *[behavior]*.	Stongly disagree 1 ☐ 2 ☐ 3 ☐ 4 ☐ 5 ☐ 6 ☐ 7 ☐ Stongly agree	
11. I have the ability to *[behavior]*.	Stongly disagree 1 ☐ 2 ☐ 3 ☐ 4 ☐ 5 ☐ 6 ☐ 7 ☐ Stongly agree	
12. Overall, I think that for me *[behavior]* would be:	Harmful 1 ☐ 2 ☐ 3 ☐ 4 ☐ 5 ☐ 6 ☐ 7 ☐ Beneficial	

[behavior]* **to be adapted for each CPD activity according to the objectives proposed for the activity**

The CPD reaction questionnaire development was funded by a Partnership for Health System Improvement grant from the Canadian Institutes of Health Research (CIHR; 2010-2013; grant # 200911PHE-216868-PHE-CFBA-19158) and by the Ministère de la Santé et des Services Sociaux du Québec (MSSS), QC, Canada.

The CPD Reaction Questionnaire ©2013 Université Laval
