# Supplementary material for: Factors Associated With Specialists’ Intention to Adopt New Behaviors After Taking Web-Based Continuing Professional Development Courses: Cross-sectional Study
Source: JMIR Med Educ. 2022 Jun 2;8(2):e34299. doi: 10.2196/34299 (PMC9204572; doi:10.2196/34299)
Supplement: Multimedia Appendix 2 [file mededu_v8i2e34299_app2.pdf]

CPD course	1			2			3			4			5		
Number of respondents, n (%)	53 (20)			44 (17)			63 (24)			61 (24)			38 (15)		
	**β**	**95% CI**	**p-value**	**β**	**95% CI**	**p-value**	**β**	**95% CI**	**p-value**	**β**	**95% CI**	**p-value**	**β**	**95% CI**	**p-value**
**Bivariate regression analysis**															
Social influences	0.44	0.13-0.75	0.006	0.34	0.13-0.56	0.0028	0.61	0.35-0.88	<.0001	0.24	-0.05-0.54	0.1065	0.40	0.22-0.58	<.0001
Beliefs about capabilities	0.94	0.64-1.25	<.0001	0.83	0.57-1.09	<.0001	1.18	0.97-1.39	<.0001	1.12	0.97-1.26	<.0001	0.64	0.49-0.80	<.0001
Moral norm	0.67	0.22-1.12	0.005	0.65	0.38-0.91	<.0001	0.65	0.38-0.93	<.0001	0.97	0.89-1.04	<.0001	0.99	0.83-1.15	<.0001
Beliefs about consequences	0.86	0.62-1.10	<.0001	0.74	0.57-0.91	<.0001	0.76	0.52-1.00	<.0001	0.55	-0.05-1.15	0.0734	0.88	0.64-1.12	<.0001

**Multivariate regression analysis**															
Social influences	0.10	-0.16-0.36	0.4342	0.005	-0.15-0.16	0.9476	-0.04	-0.23-0.15	0.6804	-0.07	-0.18-0.03	0.1506	0.08	-0.05-0.20	0.2070
Beliefs about capabilities	0.43	-0.21-0.88	0.0614	0.31	0.001-0.62	0.0492	0.80	0.54-1.06	<.0001	0.37	0.15-0.59	0.0012	0.11	-0.12-0.35	0.3238
Moral norm	-0.06	-0.49-0.37	0.7745	0.18	-0.10-0.46	0.1905	0.26	0.09-0.43	0.0029	0.71	0.55-0.87	<.0001	0.74	0.48-1.01	<.0001
Beliefs about consequences	0.58	0.23-0.92	0.0017	0.54	0.36-0.73	<.0001	0.39	0.22-0.57	<.0001	0.09	-0.07-0.26	0.2585	0.07	-0.21-0.36	0.6037

* CPD: Continuing Professional Development
** Course topic 1) to adapt the frequency of cytological exams for gynecological patients 25-45 years old to new human papillomavirus recommendations; 2) to use recommended lung cancer treatment and monitoring algorithms; 3) to use a systematic leadership approach in community health endeavors (case: preventing suicides from a bridge in Montreal); 4) to respect best practices in record keeping; and 5) to identify patients who meet the criteria for identifying a potential organ donor.
